# Blood virome of patients with traumatic sepsis

**DOI:** 10.1186/s12985-023-02162-4

**Published:** 2023-09-01

**Authors:** Qingqing Mao, Ying Liu, Ju Zhang, Wang Li, Wen Zhang, Chenglin Zhou

**Affiliations:** 1 Clinical Laboratory Center, The Affiliated Taizhou People’s Hospital of Nanjing Medical University, Taizhou, 225300 China; 2 Department of Laboratory Medicine, School of Medicine, Jiangsu University, Zhenjiang, 212013 China; 3 Clinical Laboratory Center, Xuzhou Central Hospital, Xuzhou, 221009 China

**Keywords:** Sepsis, Wounds and injuries, Mategenomics, Blood, Virome

## Abstract

Sepsis is one of the possible outcomes of severe trauma, and it poses a dire threat to human life, particularly in immunocompromised people. The most prevalent pathogens are bacteria and fungi, but viruses should not be overlooked. For viral metagenomic analysis, we collected blood samples from eight patients with post-traumatic sepsis before and seven days after treatment. The results demonstrated that *Anellovirus* predominated the viral community, followed by *Siphoviridae* and *Myoviridae*, and that the variations in viral community and viral load before and after treatment were not statistically significant. This study allows us to investigate methods for establishing NGS-based viral diagnostic instruments for detecting viral infections in the blood of sepsis patients so that antiviral therapy can be administered quickly.

**Supplementary Information:**

The online version contains supplementary material available at 10.1186/s12985-023-02162-4.

## Introduction

Sepsis is defined as “a life-threatening organ malfunction induced by a dysregulated host response to infection” [1, 2]. It is a common complication of severe burns, numerous traumas, and other severe infections. Extensive tissue damage resulting from severe trauma induces a robust inflammatory response, and an imbalance between inflammatory and anti-inflammatory cytokines disrupts immune system homeostasis, resulting in sepsis and multiple organ dysfunction syndrome (MODSZ) [3, 4]. It accounts for 10% of trauma-related deaths and 25.8% of intensive care unit (ICU) mortality [5, 6]. Post-traumatic infections that result in sepsis can be caused by any organism capable of multiplication, with bacterial and fungal infections being the most prevalent, but viremia also exists. Depending on their experience, clinicians administer broad-spectrum antibiotics as soon as sepsis is identified clinically. Numerous studies have demonstrated that the rapid administration of antimicrobial drugs that target the underlying pathogen can considerably enhance patient care and survival [7–9], but conventional diagnostic approaches do not permit rapid pathogen diagnosis. Blood culture is the gold standard for diagnosing pathogens in clinical practice, but it is time-consuming, and some bacteria may go undetected due to a variety of factors. Viral infections that cause sepsis cannot be detected by blood culture. In addition, it has recently been considered whether a patient's underlying viral infection can be a factor in the treatment failure of sepsis [10, 11]. It has been shown that patients with sepsis who have an underlying herpes virus develop viremia during the course of the disease, even if their immune system was healthy prior to the advent of sepsis, and that mortality rates are higher in patients with multiple concurrent viral infections [12]. Therefore, rapid pathogen screening of patients is essential.

Metagenomic next-generation sequencing (mNGS) permits rapid and accurate detection of possible pathogens in a variety of samples and is a potent diagnostic tool for infections. Recent studies have demonstrated that mNGS has a higher positive identification rate than blood cultures for identifying bacteria in suspected and confirmed cases of sepsis [13–15]. Whether the pathogen is a virus or bacteria that was not detected by blood culture due to a low load, this method can improve the detection rate and eradicate the issue of only being able to administer a therapy based on experience. Most current studies used mNGS to detect infection to compare its accuracy with blood culture, but paid little attention to the potential virus in the patient and even less focused on evaluating the change of virus in the body before and after treatment. In this study, we collected blood samples from eight patients with sepsis at the time of hospitalization and after 7 days of treatment. We then used mNGS to detect viral community information in the blood samples to determine whether the patients had viral infections in their bodies and the changes in viral levels before and after treatment. This investigation may contribute to the development of an NGS-based diagnostic instrument for detecting viral infections in the blood of sepsis patients so that antiviral therapy can be administered promptly.

## Patients and methods

### Sample collections

Blood samples were collected in the affiliated hospital of Jiangsu University from eight patients diagnosed with sepsis due to trauma in 2018. The inclusion criteria were patients diagnosed with systemic inflammatory response syndrome concerning international diagnostic criteria for sepsis. Each patient provided two blood samples at two distinct times, before and seven days after therapy, for a total of 16 samples. The samples were preprocessed according to our laboratory's previous protocol [16, 17], and each sample was formed into a separate library, resulting in a total of sixteen libraries. The ethics committee of Jiangsu University authorized the study.

### Library construction and sequencing

The total nucleic acids of sixteen pools (DNA and RNA) were respectively extracted using QIAamp Viral RNA Mini Kit (QIAGEN) according to the manufacturer's protocol. For RNA viruses, reverse transcriptase (Super-Script III, Invitrogen) was used to reverse-transcribe the nucleic acid amplified with the random hexamer primer into the first strand of cDNA, followed by large (Klenow) fragment (NEB) synthesis of the second strand of cDNA. For ssDNA viruses, ssDNA was converted to dsDNA using the Klenow reaction simultaneously. The nucleic acids were then subjected to viral metagenomic library construction as described in our previously published paper [17–19]. After constructing 16 libraries with 250 bp paired ends and double barcodes using the Nextera XT DNA Sample Preparation Kit (Illumina), they were sequenced on the HiSeq Illumina platform [19].

### Bioinformatic analyses

The 250 bp paired-end reads generated by HiSeq sequencing were debarcoded using vendor software from Illumina. Clonal reads were abandoned, and low sequencing quality tails were trimmed using Phred quality score 30 as the threshold. The cleaned reads from Illumina sequencing were assembled de novo within each barcode group using the Ensemble assembler to merge them into longer contigs. The assembled contigs, as well as singlets, were compared to an in-house viral proteome database using BLASTx with an E-value cutoff of 10^–5^ [20, 21].

### Phylogenetic analysis and data analysis

The cleaned reads were assembled to long contigs using the Geneious Prime (v2019.2.3) [22], and viral proteins found in this study were aligned using MUSCLE in MEGA-X [23] and performed phylogenetic analyses using MrBayes v3.2.7 [24]. Heatmap, Alpha diversity analysis, and principal coordinate composition (PCoA) were performed using R v4.1.1. The differences in viral content before and after treatment were calculated by GraphPad Prism 7.02. *p* values < 0.05 was considered statistically significant.

## Result

### Comparison of the difference in viruses before and after treatment

**Fig. 1:**
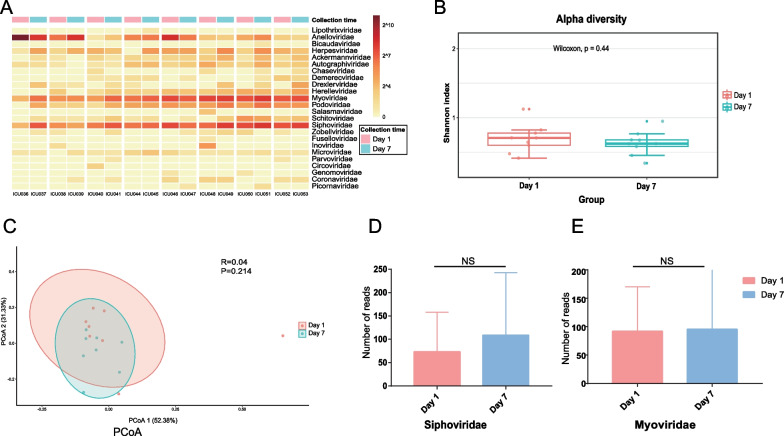
A comparison of viral variants prior to and after the therapy. **A** Heat map displaying representative viral families from sixteen pools. The names of the columns at the bottom of the graph represent pool numbers. (The data has been standardized.) The red bar at the top of the graph represents blood samples collected prior to treatment, while the blue bar represents blood samples collected seven days after therapy. The row labels on the graph’s right side correspond to the names of viral gates. The reads are log-transformed with log2 as the base, and the figure legend is located in the upper right corner. **B** Alpha diversity between the two groups (Shannon index). The horizontal bars within boxes indicate the medians. The 75th and 25th percentiles are shown by the top and bottom boxes, respectively. The top and lower whiskers extend to data within 1.5 the interquartile range of the box’s upper and lower edges, respectively. The Days are denoted by their respective colors (see color legend). **C** Principal coordinates (PCoA) analysis. Based on the Bray–Curtis ecological distance matrix, the PCoA analysis reveals disparities in species composition. **D**, **E** Wilcoxon signed-rank test of Siphoviridae and Myoviridae. Red indicates the number of reads prior to therapy, whereas blue indicates the number of reads after seven days of treatment. All analyses with *p* values 0.05 were statistically significant

Eight patients with sepsis were selected for this investigation, and 16 blood sample libraries were established by collecting blood samples again on the day of hospitalization and 7 days after treatment. After human genomic sequences were removed, these 16 libraries yielded a total of 5,809,742 reads, of which only 33,338 were corresponded to the viral genomes (Additional file 1: Supplementary Table 1). The viral readings were categorized into 25 viral families at the family level (Fig. 1A). *Anelloviridae*, *Siphoviridae*, and *Myoviridae* were well represented in all libraries. *Anelloviridae* had the highest number of reads among eukaryotic viruses, while *Siphoviridae* and *Myoviridae* had the highest proportion of reads among phages. To investigate whether the composition of viral communities changed significantly before and after treatment, we performed an Alpha diversity analysis, which indicates community richness, and a Beta diversity analysis, which indicates community variability. According to these analyses, there was no statistically significant difference (*p* > 0.05) between the viral populations before and after treatment (Fig. 1B and C).

All cases of post-traumatic sepsis in this study were caused by bacteria. Phages exert phagocytosis and other functions on bacteria, and many studies regard phages as potential alternatives to antibiotics and phage therapy as a potential solution to treat infections with multidrug-resistant pathogens. Therefore, the changes in phage content before and after treatment are worthy of our attention. We performed paired nonparametric tests on the pre- and post-treatment content changes of the two high-read phages that emerged in this study. However, the results did not reveal any statistically significant differences (Fig. 1D and E). These studies revealed no statistically significant variations between the pre- and post-treatment viral groups or viral loads among the patients in this experiment.

### *Anelloviruses* found in the study

**Fig. 2:**
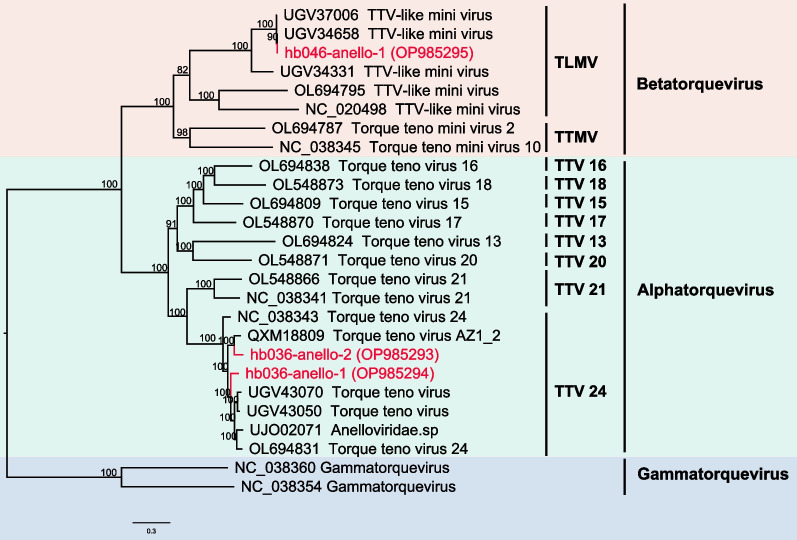
Phylogenetic analysis of Anellovirus sequences. Phylogenetic relationship of Anelloviruses based on the amino acid sequences of ORF1. Nodes with bootstrap values greater than 70 are logged. The red sequences represent the sequences obtained in this investigation

We constructed three *Anelloviruses* in the blood of two patients, one complete and two nearly complete (Additional file 2: Supplementary Table 2). *Aneloviruses* are small, single-stranded, circulating DNA viruses frequently detected in various human samples; however, there is no evidence relating them to human disease. The lengths of the three obtained sequences, numbered hb036-anello-1, hb036-anello-2, and hb046-anello-1, range between 2952 and 3721 bp. These three viruses shared between 87.37 and 99.73% identity with viral proteins uploaded to GenBank, according to a BLASTp search. *Anelloviruses* are genetically heterogeneous, with two viruses in the same individual sharing 88% similarity but not identical. Using the ORF1 region of *Anellovirus*, we constructed a phylogenetic tree that revealed two viruses belonged to *Torque teno virus 24* (TTV) and one virus clustered with *Torque teno mini virus* (TLMV) (Fig. 2). The discovered viruses most closely resembled those already uploaded to the database in the blood of infants with unexplained fever who had a high proportion of *Anellovirus* positive. In this study, the viral load of *Anellovirus* in both patients' blood decreased following therapy; however, the decline was not statistically significant due to the small sample size.

## Discussion

In this investigation, we employed mNGS to analyze the composition of the blood virus community and its alterations in patients with severe trauma-induced sepsis before and after treatment. Sepsis may result in functional immunosuppression, leading to viral reactivation and the development of viremia, such as CMV viremia [25–28]. Patients with latent virus in the body are more likely to experience viral reactivation [29]; therefore, it is essential to identify the patient's latent virus prior to treatment. In this study, the pre-treatment viral community composition had relatively high reads for *Anelloviridae*, *Siphoviridae*, and *Myoviridae*, with *Anelloviridae* dominating the blood virome. Moreover, the composition of this community is very similar to that of the healthy population [30, 31]. After seven days of treatment, blood samples were also collected and tested for viral communities in this study. Before and after treatment, there were no statistically significant differences between the composition of the viral community and the amount of virus in the blood of the eight patients. Viruses were detected at high concentrations in plasma and blood, indicating active viral replication [32]. During the seven-day treatment period, none of the eight patients took any antiviral medication, indicating that none had viremia and no viral infections or outbreaks of viral reactivation occurred.

By using de novo assemble and map, we were able to splice three nearly complete *Anelloviruses* from the blood of two individuals. It has been discovered that *Anellovirus* dominates the population of blood viruses, which is compatible with the results of this experiment [30]. Two of the three viruses we discovered were TTV, while the remaining was TTMV. *Anellovirus* cannot be linked to disease at this time because it is also present in large numbers in healthy individuals; however, multiple studies have demonstrated that the amount of the TTV viral load reflects the patient's immunity and that the TTV viral load is a useful surrogate marker of immunity. The lower the patient's immunity, the higher the TTV viral titer in the body. In this study, two patients had virtually no *Anellovirus* in their bodies prior to treatment, and their viral titers decreased after seven days of treatment, which may indicate that the treatment was effective and led to physical recovery. However, the numbers of samples were insufficient for statistical significance, and additional samples are required to verify this claim.

Many phage fragments also appeared in this study, and phages can play the role of phagocytosis of bacteria. Many studies treat phages as a potential substitute for antibiotics in treating sepsis, and some speculate that there may be a possibility of bacterial emergency activation of lysed phages during sepsis [33]. However, there was no statistically significant difference in comparing phage levels before and after treatment in this study. The reasons for this situation are complex, and many possibilities need more research to explore in depth.

Numerous things could be improved in our study. First, the sample size is too small, and the composition and variation of the viral community in post-traumatic sepsis in this study may be biased; we need to collect more samples (for example, blood samples from healthy people, blood samples from cured patients, blood samples from uncured patients) for a more thorough study. Second, only two-time points prevented us from observing changes in the viral community during the interim periods. We need to obtain additional blood samples from multiple time points for a more accurate comparison.

Overall, this was one of the few studies to examine the blood virome of patients with post-traumatic sepsis and evaluate the variations in viral communities before and after treatment. Although none of the eight patients had viremia, it is crucial to be aware of the possibility of viral infections and prevent viral reactivation in sepsis. Unlike conventional diagnostics, viral metagenomes can detect viral infections in patients more quickly; nevertheless, standardization and contamination control must be enhanced. Through this research, we have sketched out strategies for establishing NGS-based viral diagnostic tools for detecting viral infections in the blood of sepsis patients in order to facilitate timely antiviral therapy.

### Supplementary Information

**Additional file 1.** Summary of sample information of sixteen pools.

**Additional file 2.** Viral sequences identified in our study.

## Data Availability

The data that support the findings of this study are available in NCBI under the BioProject accession number PRJNA908780. The genome sequences of novel viruses were deposited to Genbank under accession number OP985293- OP985295.

## Abbreviations

MODSZ: Multiple organ dysfunction syndrome
ICU: Intensive care unit
mNGS: Metagenomic next-generation sequencing
BLAST: Basic local alignment search tool
PCoA: Principal coordinate composition
TTV: Torque teno virus
TLMV: Torque teno mini virus
